# IDP-denovo: *de novo* transcriptome assembly and isoform annotation by hybrid sequencing

**DOI:** 10.1093/bioinformatics/bty098

**Published:** 2018-02-23

**Authors:** Shuhua Fu, Yingke Ma, Hui Yao, Zhichao Xu, Shilin Chen, Jingyuan Song, Kin Fai Au

**Affiliations:** 1 Department of Internal Medicine, University of Iowa, Iowa City, USA; 2 Institute of Medicinal Plant Development, Chinese Academy of Medical Sciences, Peking Union Medical College, Beijing, China; 3 Department of Biostatistics, University of Iowa, Iowa City, USA

## Abstract

**Motivation:**

In the past years, the long read (LR) sequencing technologies, such as Pacific Biosciences and Oxford Nanopore Technologies, have been demonstrated to substantially improve the quality of genome assembly and transcriptome characterization. Compared to the high cost of genome assembly by LR sequencing, it is more affordable to generate LRs for transcriptome characterization. That is, when informative transcriptome LR data are available without a high-quality genome, a method for *de novo* transcriptome assembly and annotation is of high demand.

**Results:**

Without a reference genome, IDP-denovo performs *de novo* transcriptome assembly, isoform annotation and quantification by integrating the strengths of LRs and short reads. Using the GM12878 human data as a gold standard, we demonstrated that IDP-denovo had superior sensitivity of transcript assembly and high accuracy of isoform annotation. In addition, IDP-denovo outputs two abundance indices to provide a comprehensive expression profile of genes/isoforms. IDP-denovo represents a robust approach for transcriptome assembly, isoform annotation and quantification for non-model organism studies. Applying IDP-denovo to a non-model organism, *Dendrobium officinale*, we discovered a number of novel genes and novel isoforms that were not reported by the existing annotation library. These results reveal the high diversity of gene isoforms in *D.officinale*, which was not reported in the existing annotation library.

**Availability and implementation:**

The dataset of *Dendrobium officinale* used/analyzed during the current study has been deposited in SRA, with accession code SRP094520. IDP-denovo is available for download at www.healthcare.uiowa.edu/labs/au/IDP-denovo/.

**Supplementary information:**

Supplementary data are available at *Bioinformatics* online.

## 1 Introduction

As the new generation sequencing technologies bring substantial advances in exploring transcriptomes, a wealth of relevant bioinformatics methods, such as splice detection and transcript reconstruction, have been developed and used widely in various species (Grabherr *et al.*, 2011; Pertea *et al.*, 2015; Schulz *et al.*, 2012; Trapnell *et al.*, 2010). In particular, transcriptome analysis of model organisms has achieved tremendous progress (Darmanis *et al.*, 2015; Gonzalez-Porta *et al.*, 2013; Su *et al.*, 2002; Tang *et al.*, 2009), because their well-established reference genomes considerably reduce the problem complexity and thus improve the accuracy of sequencing data analysis (Au *et al.*, 2013; Oliver *et al.*, 2016). In contrast to the limited number of model organisms, there are hundreds of thousands of non-model organisms that are crucial to broad research areas, such as ecology, microbiology, evolutionary biology and agricultural sciences (da Fonseca *et al.*, 2016; Ekblom and Galindo, 2011). For example, a traditional Chinese medicine plant, *Dendrobium officinale*, shows drought resistance and therapeutic effects (Yan *et al.*, 2015). Research on venom gland transcriptomes of painted saw-scale viper, *Echis coloratus*, paved the way for antivenom manufacture (Hargreaves and Mulley, 2015). However, *de novo* genome assembly of non-model organisms is particularly expensive and computationally intense (Meyer *et al.*, 2015; Rokas and Abbot, 2009). Moreover, the assembled genomes are not as accurate and complete as those of model organisms, which have been polished over the years. As the cost of transcriptome sequencing of non-model organisms is affordable by all-size laboratories (Wang *et al.*, 2009), plenty of bioinformatics efforts have been made to perform *de novo* transcriptome assembly (Grabherr *et al.*, 2011; Robertson *et al.*, 2010; Schulz *et al.*, 2012).

*de novo* transcriptome assembly based on Second Generation Sequencing (SGS) short reads (SRs) is a general approach to investigate non-model organisms (Chen *et al.*, 2011; Surget-Groba and Montoya-Burgos, 2010), albeit current progress is still far from satisfying. Most SGS-based methods build de Bruijn graph of *k*-mers extracted from SRs and search for paths representing transcripts with heuristics or customized criteria (e.g. coverage) (Chang *et al.*, 2015; Fu *et al.*, 2014; Grabherr *et al.*, 2011; Luo *et al.*, 2015; Pevzner *et al.*, 2001; Simpson *et al.*, 2009; Zerbino and Birney, 2008). These methods are limited by the SR length of SGS data in a few ways: (i) transcript assembly contains numerous ambiguities or even fails in homologous or repetitive regions; (ii) accuracy of transcript assembly is called into question when a gene exhibits complex isoform expression; (iii) transcripts with low expression levels are challenging to be reconstructed, especially when certain *k*-mers are missing due to low abundance; (iv) long *k*-mers require high computing intensity whereas short *k*-mers yield high false-positive assemblies (Miller *et al.*, 2010).

In contrast to the 50–300 bp read length generated from SGS platforms, Third Generation Sequencing (TGS), including Pacific Biosciences (PacBio) (English *et al.*, 2012; Rhoads and Au, 2015; Tilgner *et al.*, 2015) and Oxford Nanopore Technologies (ONT) (Laver *et al.*, 2015; Oikonomopoulos *et al.*, 2016), produces much longer reads (1–100 kb). TGS long reads (LRs) have been exploited successfully to identify gene isoforms from human by genome-alignment-based methods, as they can cover long stretches of sequences, up to full-length transcripts (Au *et al.*, 2013; Sharon *et al.*, 2013; Tilgner *et al.*, 2014; Weirather *et al.*, 2015). Owing to the lack of reference genomes for non-model organisms, a novel assembly method is required to utilize TGS LRs that merely cover fragments of extremely long transcripts. In addition, the high error rate of TGS limits the accuracy of assembly, and the low throughput and sequencing bias of TGS render it inadequate for statistical estimation of isoform abundance.

Hybrid sequencing (‘Hybrid-Seq’) has emerged as a novel approach to integrate TGS and SGS data, in order to address the limitations of SGS-alone and TGS-alone data analyses. It can improve the overall performance and accuracy of the output data. Indeed, a series of bioinformatics tools for Hybrid-Seq transcriptome data, including LSC, IDP, IDP-fusion and IDP-ASE, have been demonstrated to elucidate transcriptomes of model organisms at the isoform level with high precision and sensitivity (Au *et al.*, 2012, 2013; Deonovic *et al.*, 2016; Weirather *et al.*, 2015). The high-quality transcript assembly by Hybrid-Seq will guarantee the accuracy of annotation and quantification of isoforms.

Here we present IDP-denovo (www.healthcare.uiowa.edu/labs/au/IDP-denovo/), a novel method to perform *de novo* transcriptome assembly by Hybrid-Seq data, and further annotate gene isoform structures and alternative splice sites without requiring a reference genome, followed by isoform abundance estimation from sequencing coverage. Using the human Hybrid-Seq transcriptome data from a lymphoblastoid cell line [GM12878 (Tilgner *et al.*, 2014)] as a gold standard, we show the superior performance of full-length transcript assembly by IDP-denovo, demonstrating the advantages of LR inclusion in comparison to existing SR-alone methods, and the advantages of its conceptual design in comparison to existing Hybrid-Seq methods (Grabherr *et al.*, 2011; Roulin *et al.*, 2014). After assembly, isoforms are clustered and annotated to show high accuracy of identifying alternative exon usage. In addition, isoform abundance is estimated by SRs and LRs. To demonstrate the utility of IDP-denovo to non-model organisms, we apply it to *D.officinale* as a proof-of-concept study and compare the results with the existing annotation library. IDP-denovo discovers 7831 novel genes that are missed by the existing annotation library, which is likely due to the complexity of gene sequences or the poor quality of genome assembly in the previous studies.

## 2 Materials and methods

### 2.1 Overview of IDP-denovo

**Fig. 1.**
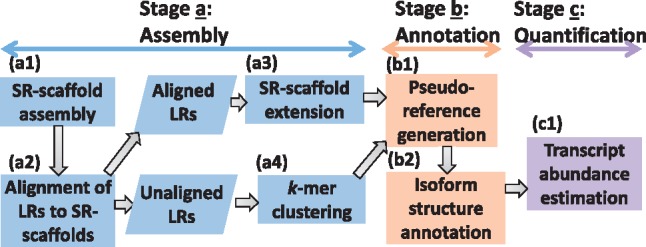
Flowchart of IDP-denovo. There are three stages in IDP-denovo: assembly, annotation and quantification. (**a**) Assembly: SRs are assembled into SR-scaffolds, followed by extension and clustering with aligned LRs. The unaligned LRs are clustered on *k*-mers. (**b**) Annotation: pseudo-references containing all expressed exonic regions are generated, and assembled transcript sequences are aligned to the pseudo-reference sequences to annotate isoform structures and identify alternative exon usage. (**c**) Quantification: the transcript abundance is estimated by LR and SR coverage based on the annotation identified in the ‘annotation’ stage

To characterize transcriptomes that lack a reference genome, IDP-denovo was designed with three stages: (i) assembly, (ii) annotation and (iii) quantification (Fig. 1). In the ‘assembly’ stage, SRs are assembled by an existing SR-alone method to generate SR-assembled scaffolds (denote as SR-scaffolds) (Fig. 1, step a1). Next, the LRs that are aligned to SR-scaffolds (Fig. 1, step a2), are used to extend the SR-scaffolds and grouped with SR-scaffolds according to locus information provided by the SR-assembly method (Fig. 1, step a3). The unaligned LRs are clustered together based on *k*-mers (Fig. 1, step a4). Using all exonic regions of transcript sequences in each cluster obtained in the ‘assembly’ stage, the ‘annotation’ stage generates longer consensus sequences with higher accuracy, named as pseudo-reference sequences (Fig. 1, step b1). Next, the transcript sequences are aligned to the pseudo-reference sequences to annotate isoform structures, including identification of alternative usage of exons and splice sites (Fig. 1, step b2). Finally, the ‘quantification’ stage estimates isoform abundance using two indices: (i) SR coverage deconvolved by the existing annotation-based statistical approach (Au *et al.*, 2013), and (ii) the number of supporting LRs (Fig. 1, step c1).

### 2.2 Transcript assembly by Hybrid-Seq data

#### *2.2.1* SR-scaffold assembly and SR-scaffold extension

**Fig. 2.**
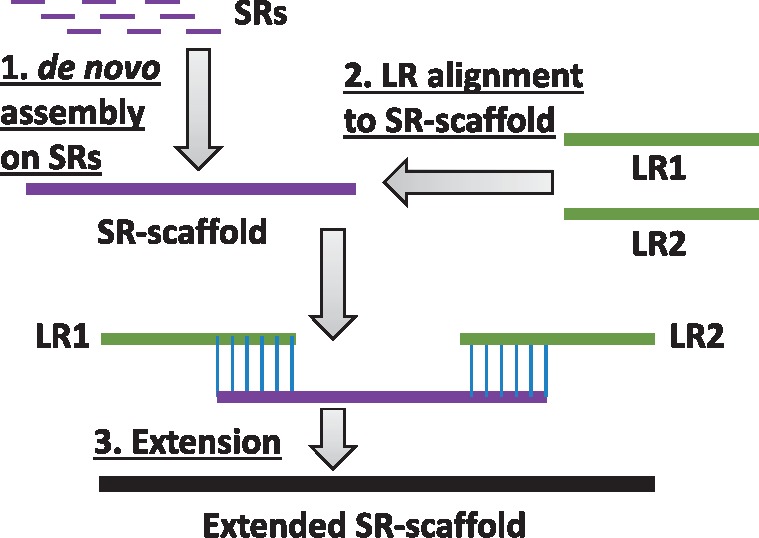
Schematic illustration of SR-scaffold extension by LRs. SR-scaffolds are generated by *de novo* assembly from SRs. Next, LRs are aligned to SR-scaffolds and then the SR-scaffolds are extended by LRs

Firstly, SRs are assembled into SR-scaffolds by a *de novo* assembly algorithm [e.g. Velvet + Oases (Schulz *et al.*, 2012; Zerbino and Birney, 2008)] (Fig. 1, step a1). However, a number of SR-scaffolds are still not long enough to cover the full length of very long transcripts (Fig. 2). The missing regions can be covered by LRs, so SR-scaffold extension by LRs is subsequently performed to get longer transcript sequences. Following error correction using SRs [e.g. by LSC (Au *et al.*, 2012)], LRs are aligned to SR-scaffolds by GMAP (Wu and Watanabe, 2005) (Fig. 1, step a2). If the alignment of a LR covers one end of the SR-scaffold and also contains an overhang region, the overhang region from the LR is used in SR-scaffold extension (Fig. 2). When more than one LR contributes to extension of the same end of a SR-scaffold, a consensus sequence of these overhang fragments will be generated by an existing consensus generation algorithm, CAP3 (Huang and Madan, 1999) to extend the SR-scaffolds.

#### *2.2.2* Clustering of SR-scaffolds and LRs

**Fig. 3.**
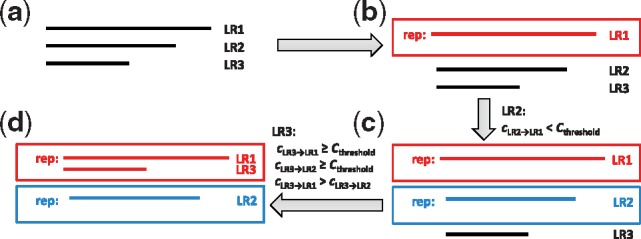
Illustration of *k*-mer-based clustering method. (**a**) Sort LRs by descending lengths. (**b**) Generate the first cluster with the longest LR (LR1) and take it as the representative LR. Then, compute the percentages of shared *k*-mers between the representative LRs from existing clusters and the following LRs sequentially. If the highest percentage is below the customized threshold *C*_threshold_, a new cluster is formed with the LR as its representative, such as LR2 (**c**). If the percentages are above the customized threshold *C*_threshold_, then assign the LR to the cluster with the highest percentage, such as LR3 (**d**)

After extension, SR-scaffolds and LRs are grouped according to the locus information provided by the SR-assembly method. Some LRs are not aligned to SR-scaffolds, as they are from genes that are not covered by SR data, missed by SR assembly, or due to misassembly by SRs, in addition to the high error rates of LRs. To rescue the important splicing information and isoforms, the unaligned LRs are clustered by a *k*-mer-based clustering approach (Fig. 1, step a4 and Fig. 3). Each cluster represents a gene, although gene families or pairs of expressed parent and pseudogenes may be represented in a single cluster. LR sequences are processed with homopolymer compression before clustering, in order to increase sensitivity and reduce computing intensity (Au *et al.*, 2012). Next, similar to CD-HIT (Li *et al.*, 2001), LRs are clustered sequentially according to the descending order of their lengths. Initially, the longest LR is assigned to the first cluster and it is set as the representative LR of this cluster. While CD-HIT computes similarity between two sequences by alignment, our *k*-mer clustering method computes their similarity by the percentage of shared *k*-mers. When considering the next LR, we examine the percentages of shared *k*-mers between this LR and the representative LRs of all existing clusters. If no existing cluster has the shared percentage of *k*-mers above a customized threshold, a new cluster is generated with this LR as the representative LR. Otherwise, this LR is assigned to the cluster with the highest percentage of shared *k*-mers. The time complexity of the clustering step is $O\left(N^{2}l\right)$, where N is the number of unaligned LRs and l is the average length of those LRs (see details in Supplementary Material: Note 1). To accelerate the clustering process, bloom filters are used to store and query *k*-mers (Melsted and Pritchard, 2011). A bloom filter is a space-efficient data structure with constant time of data addition or query at the expense of a low false positive rate (Kirsch and Mitzenmacher, 2006). The consensus sequences of SR-scaffolds and LRs in each cluster will be generated in the ‘Annotation of gene isoform structures’ stage.

### 2.3 Annotation of gene isoform structures

#### *2.3.1* Generation of pseudo-references of exonic regions

**Fig. 4.**
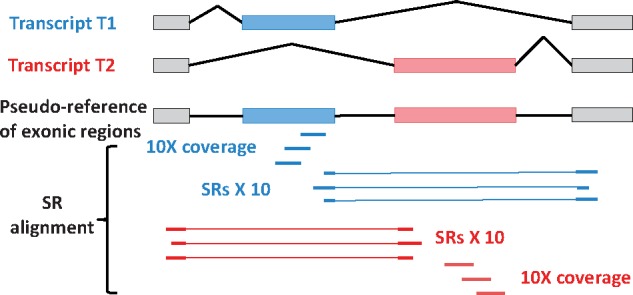
Schematic illustration of the pseudo-reference generation and SR-alignment confirmation. The pseudo-reference of the exonic regions is generated via mulitple sequence alignment with transcripts T1 and T2 to include all expressed exons. Next, the transcripts T1 and T2 are aligned to the pseudo-reference to identify the alternative usage of exons and splice sites. The predicted splices are confirmed by at least 10 SRs aligned to the splices and 10× SR coverage of the skipped exons

To annotate isoform structures from transcript sequences, we need to generate a pseudo-reference for each cluster, which is supposed to contain all expressed exons, via multiple sequence alignment (Fig. 1, step b1 and Fig. 4). In each cluster, IDP-denovo sorts the assembled transcript sequences by descending order of lengths. In the initial round, multiple sequence alignment is performed on the longest three sequences by Clustal Omega (Sievers *et al.*, 2011). Transcript sequences with <30% identities to the longest sequence are ignored. If multiple sequences remain, then the consensus sequence is generated from the alignment. In the next round, two following shorter transcript sequences will be added with the consensus sequence for multiple sequence alignment to generate the next consensus sequence. The loop continues until no more input sequence exists. The final consensus sequence will be denoted as the pseudo-reference (See the pseudocodes and details in Supplementary Material: Note 2).

#### *2.3.2* LR alignment to pseudo-references and SR alignment confirmation

In each cluster, the assembled transcript sequences are aligned to the pseudo-references by GMAP. If a gap with significant length (≥43 bp by default, see details in Supplementary Material: Note 3) is reported in the best alignment, IDP-denovo considers it as a possible alternative exon usage event. The gap is further confirmed as an alternative exon usage event with SR alignment to the pseudo-reference [e.g. by HISAT (Kim *et al.*, 2015)] (Fig. 1, step b2 and Fig. 4). Two criteria are applied: 1) at least 10 splice-aligned SRs support the gap, and 2) the gapped region contains at least 10× SR coverage. By identifying the alternative exon usage events, IDP-denovo annotates the corresponding isoform structures of each transcript.

### 2.4 Isoform abundance estimation

As transcript annotation is completed in the stage above, we use our previously published tool, IDP, to estimate transcript abundance by deconvolution of SR coverage (Au *et al.*, 2013), which is termed as ‘SR abundance index’. In addition, the number of supporting LRs for each transcript is termed as ‘LR abundance index’ (Fig. 1, step c1).

Overall, IDP-denovo outputs the assembled transcript sequences, splice sites of alternative exon usage events and two transcript abundance indices.

## 3 Results

### 3.1 Full-length gene isoform assembly and accuracy of assembled transcript sequences

We evaluated the performance of IDP-denovo using the human GM12878 dataset (See Supplementary Material: Note 4), since the high-quality human genome and gene annotation library can be used as a gold standard in performance evaluation. The first step of IDP-denovo is to assemble SR-scaffolds (Fig. 1, step a1). In order to obtain the best SR-scaffolds, we evaluated the performance of five existing SR-alone methods, including Trinity (Grabherr *et al.*, 2011), SOAPdenovo-Trans (Xie *et al.*, 2014), Bridger (Chang *et al.*, 2015), Trans-ABySS (Robertson *et al.*, 2010) and Velvet + Oases (Schulz *et al.*, 2012; Zerbino and Birney, 2008). Using the GM12878 dataset, we measured precision-recall statistics as established by Li *et al* (Li *et al.*, 2014) (See Supplementary Material: Note 5 for the detailed parameter settings for each software and an expanded description of the statistics). Among the SR-alone methods, Trans-ABySS achieved the highest precision (0.87), while the corresponding recall was only 0.14 (Table 1). Velvet + Oases produced the highest recall (0.29). Using the *F_1_* score to evaluate the overall performance of both precision and recall, Velvet + Oases had the best performance (*F_1_* score = 0.42) among the five SR-alone methods. Therefore, we used Velvet + Oases to assemble SR-scaffolds in IDP-denovo.

**Table 1.** Comparison of IDP-denovo with existing methods

Evaluation metrics	SR-alone assembly methods					Hybrid assembly methods		
	Trinity	SOAPdenovo-Trans	Bridger	Trans-ABySS	Velvet+ Oases	Trinity	Roulin’s pipeline	IDP-denovo
**Precision**	0.76	0.78	0.65	0.87	0.77	0.77	0.77	***0.92***
**Recall**	0.17	0.16	0.21	0.14	0.29	0.17	0.24	***0.40***
***F_1_* score**	0.28	0.27	0.32	0.24	0.42	0.28	0.37	***0.56***

*Note*: The best performance of SR-alone assembly methods is underlined. The best performance among all methods is bold, underlined and italic.

**Fig. 5.**
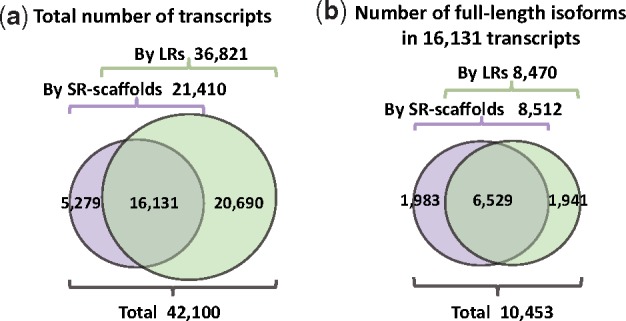
The Ensembl-annotated gene isoform identification by SR-scaffolds and/or LRs. (**a**) 16 131 gene isoforms are covered by both SR-scaffolds and LRs, while 5279 are only covered by SR-scaffolds and 20 690 only covered by LRs. (**b**) The distribution of 10 453 Ensembl-annotated full-length gene isoforms identified among 16 131 gene isoforms (shown in a) detected by both SR-scaffolds and LRs

Next, we sought to improve transcript detection by adding LRs into SR-scaffolds. LRs covered 36 821 transcripts, including 20 690 transcripts that were missed by SR-scaffolds (Fig. 5a). It may be attributed to misassembly of SR-scaffolds in repetitive regions or problematic reconstruction of lowly expressed transcripts as well as no coverage in SR data. Remarkably, the transcripts missed by SR-scaffolds but covered by LRs show significantly lower abundance than those covered by SR-scaffolds (*P*-value < 2.2e-16, see details in Supplementary Material: Note 6). SR-scaffolds covered a total of 21 410 transcripts, with 5279 transcripts that were not found by LRs. This is likely due to the sequencing bias of TGS. Therefore, pooling LRs with SR-scaffolds by IDP-denovo recovers the transcripts missed by either SR-alone or LR-alone method, and thus improves sensitivity.

In addition, 16 131 transcripts, including 10 453 full-length isoforms, were detected by either SR-scaffolds or LRs (Fig. 5b). While the vast majority of full-length isoforms were detected by both SR-scaffolds and LRs (6529 transcripts), 1983 transcripts were detected only by SR-scaffolds and 1941 were detected only by LRs (Fig. 5b). These data demonstrate the importance of integrating LRs and SR-scaffolds in order to maximize the number of recovered full-length transcripts.

**Fig. 6.**
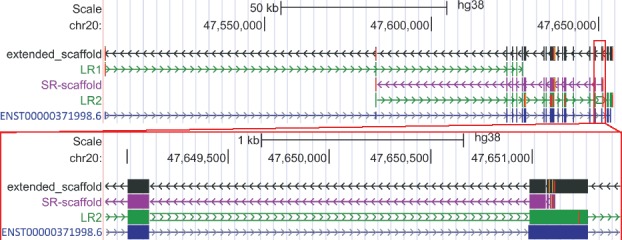
Full-length isoform of gene *AIB1* is rescued by SR-scaffold extension using LRs. Neither SR-scaffold (purple track) nor LRs (LR1 and LR2 in green tracks) can cover all splices of the isoform ENST00000371998.6 of *AIB1*. However, extended SR-scaffold with LRs covers all splices of the isoform. The zoomed-in region shows the sequencing errors (mismatches and indels) in LR2, which are corrected by the SR-scaffold by generating the consensus sequence

However, a number of transcripts were not detected as full-length isoforms by either SR-scaffolds or LRs. To assemble full-length transcripts, IDP-denovo performed SR-scaffold extension using LRs (Fig. 1, step a3). This approach recovered 702 full-length isoforms. An example from the GM12878 dataset is shown in Figure 6: the extended SR-scaffold, but not either SR-scaffold or LRs alone, recovered all annotated splice sites of the isoform ENST00000371998.6 of *AIB1*, an oncogene that is associated with drug resistance and a significant decrease in mortality (Tikkanen *et al.*, 2000).

We also evaluated the performance of IDP-denovo relative to the SR-alone methods. As compared to the results with Velvet + Oases (the superior SR-alone method), adding LRs with IDP-denovo significantly improved the precision, recall and *F_1_* score (Table 1). High SR coverage aids in assembly of accurate SR-scaffolds (step a1 in Fig. 1), while low SR coverage can lead to low-accuracy assembly that further prevents LRs from being aligned correctly to extend SR-scaffolds. In the regions uncovered by SR-scaffolds but extended by LRs, high LR coverage is helpful to generate accurate consensus from error-prone LRs (step a3 in Fig. 1). (See details in Supplementary Material: Note 7).

Therefore, integration of SR-scaffolds and LRs by IDP-denovo not only increased the total number of transcripts assembled as well as the number of full-length transcripts, but also had superior precision, recall and *F_1_* score as compared to SR-alone methods. While a caveat of LRs is a high error rate, these errors are corrected by IDP-denovo because SR-scaffolds cover the errors. In the example with *AIB1* (Fig. 6), the transcript assembled by IDP-denovo using Hybrid-Seq data contained far fewer errors than LRs alone. Thus, using Hybrid-Seq data by IDP-denovo also greatly improves the accuracy of transcript sequences assembled by correcting errors in LRs.

### 3.2 Comparison of IDP-denovo to the existing assembly methods with Hybrid-Seq data

Currently two methods have been reported for *de novo* assembly with Hybrid-Seq data: 1) a new version of Trinity (Grabherr *et al.*, 2011); and 2) a pipeline proposed by the Roulin group (Roulin *et al.*, 2014) (referred to herein as ‘Roulin’s pipeline’). We compared IDP-denovo to these two methods using parameter settings described in Supplementary Material: Note 8. Both Trinity and Roulin’s pipeline achieved a precision of 0.77, while Roulin’s pipeline gave a higher recall (0.24) and a higher *F_1_* score (0.37) than Trinity (Table 1). By contrast, IDP-denovo demonstrated better performance in terms of precision, recall and *F_1_* score (Table 1), as well as higher sensitivity [defined as the number of full-length reconstructed transcripts by Chang *et al.* (2015); Table 2; see also Supplementary Material: Note 8]. IDP-denovo reconstructed 32 393 annotated transcripts, with 16 597 as full-length transcripts, while Trinity and Roulin’s pipeline reconstructed fewer annotated transcripts (31 358 and 20 316, respectively) and full-length reference transcripts (12 698 and 8908, respectively). Trinity employs LRs to refine assembly of isoforms with complex structures by SRs, but does not use LRs to recover transcripts or transcript fragments missed by SRs. Roulin’s pipeline was designed to assemble Roche 454 LRs and Illumina SRs separately, followed by clustering and removing redundant contigs. LRs from PacBio platform (as well as ONT) are more error-prone than 454 data, so Roulin’s pipeline underperformed when PacBio data were used. Thus, IDP-denovo has enhanced performance over other existing Hybrid-Seq assembly methods.

**Table 2.** Comparison of IDP-denovo with existing hybrid methods

	Trinity (Hybrid-Seq)	Roulin’s pipeline	IDP-denovo
Reference transcripts	31 358	20 316	32 393
Full-length reference transcripts (sensitivity)^a^	12 698	8908	16 597

a The number of full-length reconstructed transcripts, defined by Chang *et al.* (2015); see ‘Supplementary Material: Note 8’.

### 3.3 Optimization of *k*-mer clustering

Our next goal is to perform annotation of transcripts. First, however, it is necessary to group together (referred to as ‘clustering’) transcripts that arise from the same gene, where each cluster contains multiple isoforms of one gene. Whereas the SR-scaffold extension process (Fig. 1, step a3) clustered the aligned LRs, a number of LRs remained unaligned. To address this, we developed a *k*-mer-based clustering approach to group unaligned LRs (Fig. 1, step a4). Two parameters influence our *k*-mer clustering method: length of *k*-mer (*k*) and percentage threshold (*C*_threshold_) of shared *k*-mers between each LR and the representative LR (see details in ‘Clustering of SR-scaffolds and LRs’ in Materials and methods section) in a cluster. To find the optimal combination of *k* and *C*_threshold_, we conducted a proof-of-concept study using 94 506 LRs from Chr19 (chromosome 19 from human genome) in GM12878 dataset as the training data. Chr19 has the highest gene density among all human chromosomes and a reasonable chromosome size (Grimwood *et al.*, 2004). We set *k* as 13, 15 and 17 and *C*_threshold_ as 0.04, 0.05 and 0.06, according to the previous studies (Aflitos *et al.*, 2015; Ghodsi *et al.*, 2011; Ondov *et al.*, 2016). Four typical measures of clustering performance were used: the Jaccard index, precision, recall and F-measure (See details in Supplementary Material: Note 9). As shown in Table 3, the optimal values of these measures were obtained when *k *= 15 and *C*_threshold_* *= 0.05. We also got the same optimal parameter setting when clustering unaligned LRs from Chr19 (See details in Supplementary Material: Note 9). Therefore, this parameter setting was subsequently used to cluster unaligned LRs.

**Table 3.** Performance of *k*-mer clustering with different combinations of lengths of *k*-mer and percentage cutoff *C*_threshold_

	Percentage cutoff *C*_threshold_	Length of *k*-mer		
		13	15	17
Jaccard index	0.04	0.955	0.955	0.965
	0.05	0.959	***0.967***	***0.967***
	0.06	0.959	***0.967***	0.964
Precision	0.04	0.993	0.995	0.994
	0.05	0.995	0.995	0.995
	0.06	0.995	0.995	***0.996***
Recall	0.04	0.961	0.960	0.970
	0.05	0.963	***0.972***	0.971
	0.06	0.964	0.971	0.968
F-measure	0.04	0.977	0.977	0.982
	0.05	0.979	***0.983***	***0.983***
	0.06	0.979	***0.983***	0.982

*Note:* Results with the best performance for each performance measure are bold, underlined and italic.

### 3.4 Completeness of exonic regions in pseudo-references

**Fig. 7.**
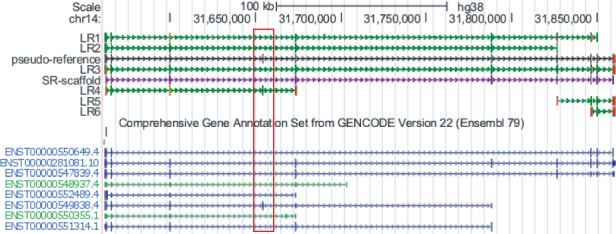
The pseudo-reference generated by IDP-denovo covers all expressed exons of the isoform of gene *NUBPL*. None of the single LRs can cover all expressed exons. An exon from the isoform ENST00000549838.4 that is highlighted in the dashed box is only covered by LR4 but not found in other LRs or the SR-scaffold. It is covered by the pseudo-reference generated by IDP-denovo

After clustering, IDP-denovo generates a longer consensus sequence termed ‘pseudo-reference of exonic regions’, which ideally covers all expressed exonic regions of a gene (Fig. 1, step b1). This step is necessary in order to perform gene isoform annotation when a reference genome is not available. Correct annotation of gene isoforms therefore requires complete exonic regions in the pseudo-references. For example, an annotated exon of isoform ENST00000549838.4 from the gene *NUBPL* on Chr14, was only found in one LR (LR4) and missed in the SR-scaffold and the longest LR of the gene (LR3) in GM12878 dataset (Fig. 7). That is, using the longest sequence in a cluster, including the longest SR-scaffold, cannot guarantee the completeness of exonic regions. However, the pseudo-reference of exonic regions generated by IDP-denovo covered all expressed exonic regions, including the ‘missing’ exon from the SR-scaffold or LR3. When IDP-denovo was applied to the whole GM12878 dataset, pseudo-references covered 158 967 out of 173 719 exons (91.51%) that were contained by the SR-scaffolds and the LRs. The uncovered exons may be partially due to error in clustering. It is possible that some transcripts were not grouped into the correct clusters and thus their specific exons were lost in the pseudo-references. Uncorrected errors in LRs could also affect the completeness of the exonic regions.

### 3.5 Accuracy of identifying alternative exon usage

**Fig. 8.**
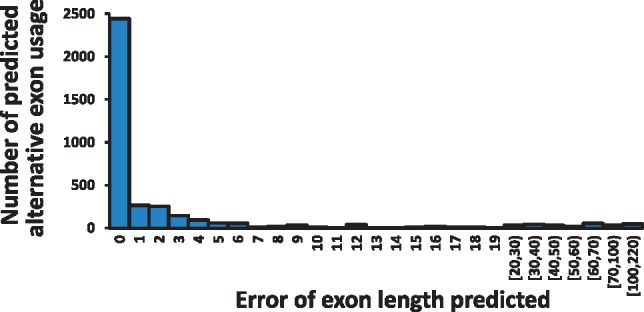
Distribution of errors of 3763 exon lengths predicted by IDP-denovo. The median is 0 bp and the mean is 6.06 bp. A small number of outliers with large errors likely result from incorrect alignments

After pseudo-references of exonic regions are generated, IDP-denovo detects alternative exon usage by aligning SR-scaffolds and LRs to the pseudo-references. When RNA sequencing data were analyzed without a reference genome, we designed a step to perform isoform annotation to identify alternative exon usage and confirm with SR alignment (Fig. 1, step b2). Errors in identifying alternative exon usage can introduce frame-shift in protein coding regions. Using the GM12878 data, we evaluated the errors by examining the difference between annotation of alternative exon usage by IDP-denovo and by aligning transcripts to the reference genome (See details in Supplementary Material: Note 10). Because of misalignment, sequencing errors or incompleteness of the reference genome, some events are difficult to be confirmed by transcript alignment to the reference genome. IDP-denovo reported 4140 alternative exon usage events, out of which 3763 (90.89%) were confirmed. As compared to the transcript alignment with the reference genome, most of the errors were 0 bp (58.99%) and a majority (85.01%) of annotations had <5 bp errors. (Fig. 8; See Supplementary Material: Note 10 for the definition of error). This suggests that IDP-denovo can identify alternative exon usage with high accuracy, which is further helpful in detecting alternative peptides. Very few outliers (155) with errors >50 bp were observed, which were likely caused by misalignments of transcripts to the pseudo-references.

### 3.6 Performance of transcript abundance estimation

A fundamental goal of transcriptome characterization is to estimate the abundance of various isoforms of a given gene. We therefore investigated the performance of IDP-denovo to quantify isoform abundance using SR or LR coverage. The SR abundance index is calculated as the RPKM, which is estimated using a Poisson model of SR coverage and maximum likelihood estimation (Au *et al.*, 2013; Jiang and Wong, 2009). The LR abundance index is calculated as the number of supporting LRs for each transcript (Fig. 1, step c1). Since some transcripts are covered by either SRs only or LRs only (Fig. 5a), the abundance estimation will fail to cover all transcripts if only one type of data is used. IDP-denovo outputs two abundance indices estimated by SR and LR data respectively, so that the abundance of all 42 100 transcripts (in Fig. 5a) can be estimated.

**Fig. 9.**
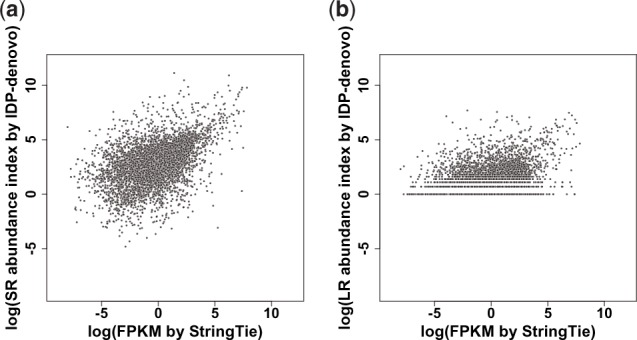
Correlation of abundance indices output by IDP-denovo and FPKM reported by StringTie (5967 isoforms). (**a**) It is a scatter plot of SR abundance index and FPKM reported by StringTie. (**b**) It is a scatter plot of LR abundance index and FPKM reported by StringTie

The reliability of two abundance indices estimated by SR and LR data can be evaluated in two ways: (i) accuracy of abundance estimation and (ii) accuracy of lowly-expressed isoform abundance estimation. To evaluate these two accuracies, we used a reference genome–based method for abundance estimation by SR coverage named StringTie (Pertea *et al.*, 2015) as the gold standard (see details in Supplementary Material: Note 11). The Spearman and Pearson correlation coefficients between SR abundance index and FPKM estimated by StringTie are 0.51 and 0.49, respectively, while those between LR abundance index and FPKM by StringTie are 0.35 and 0.35, respectively. Therefore, SR abundance index by IDP-denovo has a closer estimation of the widely-used abundance index by StringTie (Fig. 9). The incompleteness of assembly and annotation, and uncorrected sequencing errors can cause abundance estimation biases in the *de novo* transcriptome analysis. Moreover, because LR abundance index merely reports the numbers of supporting LRs, which are discrete values, it poorly estimates low abundance level. In particular, relatively low throughput and sequencing bias (e.g. size selection protocol in PacBio library preparation) in the TGS platforms further result in inaccuracy of the LR abundance index on lowly-expressed isoforms. In addition, as a LR can usually support multiple transcripts, a simple counting of supporting LRs by LR abundance index would overestimate the transcript abundance. Taken together, SR abundance index is recommended to estimate isoform abundance, while LR abundance index can be used for the isoforms that are covered only by LRs.

### 3.7 Application of IDP-denovo to a non-model organism, *D.officinale*

We further performed a proof-of-concept demonstration of IDP-denovo, with its application to a non-model organism, *D.officinale*, which is of a broad range of therapeutic effects as a Chinese medicine plant (Yan *et al.*, 2015). A draft assembly of the genome and a poor-quality annotation library of *D.officinale* were previously published (Yan *et al.*, 2015). In total, 972 412 PacBio LRs (median length is 716 bp and up to 6445 bp) and 45 013 277 paired-end Illumina SRs (2× 138 bp) were used; 321 433 LRs have been corrected with SRs.

**Fig. 10.**
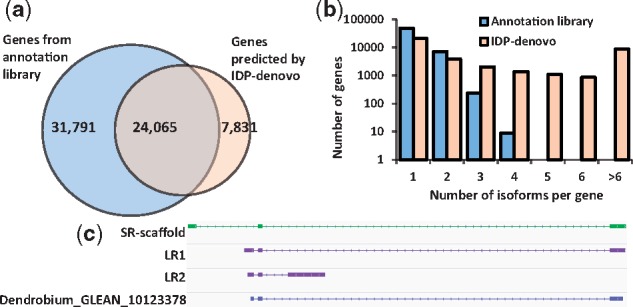
IDP-denovo reports comprehensive transcriptome characterization of *D.officinale*. (**a**) Gene identification from *D.officinale* by IDP-denovo, compared to the reference annotation library (Wu *et al.*, 2016; Yan *et al.*, 2015). IDP-denovo covers >43% (24 065) expressed genes that are reported in the reference annotation library. IDP-denovo also assembles transcripts from 7831 genes missed by the reference annotation library. (**b**) Distribution of the numbers of isoforms of annotated and novel genes reported by IDP-denovo. The high sensitivity of IDP-denovo uncovers large diversity of isoform structures. It improves the annotation library of *D.officinale*, the existing version of which mostly reports one isoform for each gene. (**c**) An example of comprehensive transcriptome characterization by IDP-denovo. Locus ‘Dendrobium_GLEAN_10123378’ is annotated with an isoform containing all splice sites found in LR1. However, the alignments of the SR-scaffold and LR2 to the locus show the expression of two other isoforms with two unannotated exons

We applied IDP-denovo to the Hybrid-Seq data from *D.officinale* (See details in Supplementary Material: Note 12). We found 7831 genes that were not reported in the existing annotation library, including 6834 aligned to the draft genome and 997 unaligned (Fig. 10a). Some annotated genes are not identified by IDP-denovo, which is probably due to (i) tissue-specific gene expression, or (ii) low gene expression, or (iii) uncorrected errors from LRs or (iv) the poor quality of the existing annotation library. Moreover, IDP-denovo discovered the high diversity of isoform expression for *D.officinale.* The genes reported in the existing annotation library are mostly annotated with single isoforms, while multiple isoform expressions were found in 17 989 genes by IDP-denovo (Fig. 10b). For example, a locus ‘Dendrobium_GLEAN_10123378’ is annotated with only a single isoform containing three exons, which is supported by a LR (LR1). However, alignment of the SR-scaffold and the other LR (LR2) to the locus shows that there are two novel isoforms with two unannotated exons (Fig. 10c). This suggests that IDP-denovo identifies more splice sites, even without a reference genome. Therefore, IDP-denovo with Hybrid-Seq data provides a comprehensive approach for characterizing the transcriptomes of non-model organisms.

## 4 Discussion

Although studies of non-model organisms play important roles in many investigations (Ekblom and Galindo, 2011), the lack of well annotated genomes enormously limits our capability of deeper understanding of their genomes, transcriptomes and gene structures. The annotated reference genome from a closely related species can aid in understanding of the non-model organism of interest, in the studies of sequence assembly (Surget-Groba and Montoya-Burgos, 2010), phylogenetic surveys (Shin *et al.*, 2012) and prediction of homologous proteins (Quistad *et al.*, 2016) etc.

In the past years, the LR sequencing technologies, such as PacBio and ONT, have been demonstrated to considerably improve the quality of genome assembly and transcriptome characterization (Au *et al.*, 2013; Bolisetty *et al.*, 2015). Compared to the high cost of genome assembly by LR sequencing, it is more affordable to generate LRs for transcriptome characterization. That is, when informative transcriptome LR data are available without a high-quality genome, a method for *de novo* transcript assembly and annotation is of high demand. IDP-denovo solves this problem with the assistance of SRs that can complement the weakness of LRs and thus improve the accuracy of assembled transcript sequences as well as the quantitative analysis. Without the requirement of a reference genome, IDP-denovo performs *de novo* transcript assembly with LR and SR data, and further annotates isoform structures and alternative usage of exons, followed by abundance estimation from sequencing coverage.

We examined the performance of IDP-denovo using the GM12878 data, since the high-quality human genome plus the reference genome-based methods can provide a reliable gold standard. IDP-denovo showed superior sensitivity and reconstructed a large number of full-length gene isoforms that were missed by the SR-alone or LR-alone approach. However, in a small number of cases that SR-scaffolds only covered fragments and no LR was generated from the gene due to low throughput or sequencing bias, IDP-denovo may not be able to assemble full-length isoforms. In addition to full-length isoform identification, IDP-denovo can produce high-quality transcript sequences through error correction of LRs and consensus sequence generation during assembly process. When we compared IDP-denovo to the existing methods using SR-alone data or the same Hybrid-Seq data, IDP-denovo had the highest precision, recall and sensitivity, and thus showed the best overall transcript assembly performance. With the assembled transcripts, IDP-denovo identifies alternative usage of exons, splice sites and gene isoforms. In order to annotate alternative splicing and gene isoforms, we applied a *k*-mer clustering approach to cluster assembled transcripts of the same gene into a single group. Although the other clustering tool ToFU is available (Gordon *et al.*, 2015), it clusters LRs from the same gene isoform, and thus is not applicable for our aim of isoform annotation. As only RNA sequencing data are used, it is not possible to identify the introns in the genome and splice sites that exist in all expressed transcripts, because no gaps corresponding to these introns and splice sites could be detected in multiple sequence alignment of transcripts. Once a reference genome is available, they can be easily identified. In the previous studies of annotating gene structure, splicing graphs have been applied to high-accuracy transcript sequences (Heber *et al.*, 2002; Santhanam and Krithivasan, 2006), while its application to error-prone LRs may introduce numerous ambiguities in splicing site prediction. Our approach works well for the vast majority of alternative exon usage events, but it is problematic for very small alternative exon usage events (<43 bp), given the difficulty of recognizing these in low-quality TGS data. IDP-denovo estimates transcript abundance based on coverage of SRs or LRs. IDP-denovo outputs two abundance indices that together cover all assembled transcripts including those missed by either abundance estimation. Abundance estimation by SR coverage may overestimate the expression level of assembled transcripts in some cases when not all transcripts are recovered. Abundance estimation with LRs may also not be sensitive and reliable for the lowly expressed transcripts.

The proof-of-concept application of IDP-denovo to *D.officinale* showed that it can provide a comprehensive transcriptome assembly and an annotation library of non-model organisms. IDP-denovo rescued a number of novel genes and transcripts that were missed by the existing annotation library, and also discovered high diversity of alternative splice sites and isoforms in *D.officinale*, which were not reported previously. With the improvement of LR sequencing technologies on sequencing errors, read length and throughput, IDP-denovo, taking advantage of strengths of LRs and SRs, will provide a powerful yet affordable approach to comprehensively characterize transcriptomes, and thus will facilitate studies of non-model organisms in the near future.

## Supplementary Material

Supplementary Data

## References

[bty098-B1] Aflitos S.A. et al (2015) Cnidaria: fast, reference-free clustering of raw and assembled genome and transcriptome NGS data. BMC Bioinformatics, 16. 10.1186/s12859-015-0806-7 PMC4630969 26525298

[bty098-B2] Au K.F. et al (2013) Characterization of the human ESC transcriptome by hybrid sequencing. Proc. Natl. Acad. Sci. USA, 110, E4821–E4830. 24282307 10.1073/pnas.1320101110 PMC3864310

[bty098-B3] Au K.F. et al (2012) Improving PacBio long read accuracy by short read alignment. PLoS One, 7, e46679. 23056399 10.1371/journal.pone.0046679 PMC3464235

[bty098-B4] Bolisetty M.T. et al (2015) Determining exon connectivity in complex mRNAs by nanopore sequencing. Genome Biol., 16. 10.1186/s13059-015-0777-z PMC4588896 26420219

[bty098-B5] Chang Z. et al (2015) Bridger: a new framework for de novo transcriptome assembly using RNA-seq data. Genome Biol., 16, 30. 25723335 10.1186/s13059-015-0596-2 PMC4342890

[bty098-B6] Chen G. et al (2011) De novo transcriptome assembly of RNA-Seq reads with different strategies. Sci. China Life Sci., 54, 1129–1133. 22227905 10.1007/s11427-011-4256-9

[bty098-B7] da Fonseca R.R. et al (2016) Next-generation biology: sequencing and data analysis approaches for non-model organisms. Mar. Genom., 30, 3–13. 10.1016/j.margen.2016.04.012 27184710

[bty098-B8] Darmanis S. et al (2015) A survey of human brain transcriptome diversity at the single cell level. Proc. Natl. Acad. Sci. USA, 112, 7285–7290. 26060301 10.1073/pnas.1507125112 PMC4466750

[bty098-B9] Deonovic B. et al (2016) IDP-ASE: haplotyping and quantifying allele-specific expression at the gene and gene isoform level by hybrid sequencing. Nucleic Acids Res., 45:e32. 10.1093/nar/gkw1076 PMC5952581 27899656

[bty098-B10] Ekblom R., Galindo J. (2011) Applications of next generation sequencing in molecular ecology of non-model organisms. Heredity, 107, 1–15. 21139633 10.1038/hdy.2010.152 PMC3186121

[bty098-B11] English A.C. et al (2012) Mind the gap: upgrading genomes with pacific biosciences RS long-read sequencing technology. Plos One, 7, e47768. 23185243 10.1371/journal.pone.0047768 PMC3504050

[bty098-B12] Fu S.H. et al (2015) Heuristic pairwise alignment of de Bruijn Graphs to facilitate simultaneous transcript discovery in related organisms from RNA-Seq data. Bmc Genomics, 16. 10.1186/1471-2164-16-S11-S5 PMC4652555 26576690

[bty098-B13] Ghodsi M. et al (2011) DNACLUST: accurate and efficient clustering of phylogenetic marker genes. BMC Bioinformatics, 12, 271. 21718538 10.1186/1471-2105-12-271 PMC3213679

[bty098-B14] Gonzalez-Porta M. et al (2013) Transcriptome analysis of human tissues and cell lines reveals one dominant transcript per gene. Genome Biol., 14, R70. 23815980 10.1186/gb-2013-14-7-r70 PMC4053754

[bty098-B15] Gordon S.P. et al (2015) Widespread polycistronic transcripts in fungi revealed by single-molecule mRNA sequencing. Plos One, 10, e0132628. 26177194 10.1371/journal.pone.0132628 PMC4503453

[bty098-B16] Grabherr M.G. et al (2011) Full-length transcriptome assembly from RNA-Seq data without a reference genome. Nat. Biotechnol., 29, 644–U130. 21572440 10.1038/nbt.1883 PMC3571712

[bty098-B17] Grimwood J. et al (2004) The DNA sequence and biology of human chromosome 19. Nature, 428, 529–535. 15057824 10.1038/nature02399

[bty098-B18] Hargreaves A.D., Mulley J.F. (2015) Assessing the utility of the Oxford Nanopore MinION for snake venom gland cDNA sequencing. PeerJ, 3, e1441. 26623194 10.7717/peerj.1441 PMC4662598

[bty098-B19] Heber S. et al (2002) Splicing graphs and EST assembly problem. Bioinformatics, 18, S181–S188. 10.1093/bioinformatics/18.suppl_1.s181 12169546

[bty098-B20] Huang X.Q., Madan A. (1999) CAP3: a DNA sequence assembly program. Genome Res., 9, 868–877. 10508846 10.1101/gr.9.9.868 PMC310812

[bty098-B21] Jiang H., Wong W.H. (2009) Statistical inferences for isoform expression in RNA-Seq. Bioinformatics, 25, 1026–1032. 19244387 10.1093/bioinformatics/btp113 PMC2666817

[bty098-B22] Kim D. et al (2015) HISAT: a fast spliced aligner with low memory requirements. Nat. Methods, 12, 357–U121. 25751142 10.1038/nmeth.3317 PMC4655817

[bty098-B23] Kirsch A., Mitzenmacher M. (2006) Less hashing, same performance: building a better bloom filter. Lect. Notes Comput. Sci., 4168, 456–467.

[bty098-B24] Laver T. et al (2015) Assessing the performance of the Oxford Nanopore Technologies MinION. Biomol. Detect. Quantif., 3, 1–8. 26753127 10.1016/j.bdq.2015.02.001 PMC4691839

[bty098-B25] Li B. et al (2014) Evaluation of de novo transcriptome assemblies from RNA-Seq data. Genome Biol., 15. 10.1186/s13059-014-0553-5 PMC4298084 25608678

[bty098-B26] Li W.Z. et al (2001) Clustering of highly homologous sequences to reduce the size of large protein databases. Bioinformatics, 17, 282–283. 11294794 10.1093/bioinformatics/17.3.282

[bty098-B27] Luo R.B. et al (2015) SOAPdenovo2: an empirically improved memory-efficient short-read de novo assembler (vol 1, 18, 2012). Gigascience, 4. 10.1186/2047-217X-1-18 PMC3626529 23587118

[bty098-B28] Melsted P., Pritchard J.K. (2011) Efficient counting of k-mers in DNA sequences using a bloom filter. BMC Bioinformatics, 12, 333. 21831268 10.1186/1471-2105-12-333 PMC3166945

[bty098-B29] Meyer W.K. et al (2015) Evolutionary history inferred from the de novo assembly of a nonmodel organism, the blue-eyed black lemur. Mol. Ecol., 24, 4392–4405. 26198179 10.1111/mec.13327 PMC4557055

[bty098-B30] Miller J.R. et al (2010) Assembly algorithms for next-generation sequencing data. Genomics, 95, 315–327. 20211242 10.1016/j.ygeno.2010.03.001 PMC2874646

[bty098-B31] Oikonomopoulos S. et al (2016) Benchmarking of the Oxford Nanopore MinION sequencing for quantitative and qualitative assessment of cDNA populations. Sci. Rep. UK, 6. 10.1038/srep31602 PMC4995519 27554526

[bty098-B32] Oliver S.G. et al (2016) Model organism databases: essential resources that need the support of both funders and users. BMC Biol., 14. 10.1186/s12915-016-0276-z PMC4918006 27334346

[bty098-B33] Ondov B.D. et al (2016) Mash: fast genome and metagenome distance estimation using MinHash. Genome Biol., 17. 10.1186/s13059-016-0997-x PMC4915045 27323842

[bty098-B34] Pertea M. et al (2015) StringTie enables improved reconstruction of a transcriptome from RNA-seq reads. Nat. Biotechnol., 33, 290. 25690850 10.1038/nbt.3122 PMC4643835

[bty098-B35] Pevzner P.A. et al (2001) An Eulerian path approach to DNA fragment assembly. Proc. Natl. Acad. Sci. USA, 98, 9748–9753. 11504945 10.1073/pnas.171285098 PMC55524

[bty098-B36] Quistad S.D. et al (2016) Using viromes to predict novel immune proteins in non-model organisms. R. Soc. B Biol. Sci., 283, 20161200 *P*10.1098/rspb.2016.1200 PMC5013795 27581878

[bty098-B37] Rhoads A., Au K.F. (2015) PacBio sequencing and its applications. Genomics Proteomics Bioinf., 13(5), 278–289. 10.1016/j.gpb.2015.08.002 PMC4678779 26542840

[bty098-B38] Robertson G. et al (2010) De novo assembly and analysis of RNA-seq data. Nat. Methods, 7, 909–U962. 20935650 10.1038/nmeth.1517

[bty098-B39] Rokas A., Abbot P. (2009) Harnessing genomics for evolutionary insights. Trends Ecol. Evol., 24, 192–200. 19201503 10.1016/j.tree.2008.11.004

[bty098-B40] Roulin A.C. et al (2014) De novo transcriptome hybrid assembly and validation in the European Earwig (Dermaptera, *Forficula auricularia*). Plos One, 9, e94098. 24722757 10.1371/journal.pone.0094098 PMC3983118

[bty098-B41] Santhanam R., Krithivasan K. (2006) Graph splicing systems. Discret. Appl. Math., 154, 1264–1278.

[bty098-B42] Schulz M.H. et al (2012) Oases: robust de novo RNA-seq assembly across the dynamic range of expression levels. Bioinformatics, 28, 1086–1092. 22368243 10.1093/bioinformatics/bts094 PMC3324515

[bty098-B43] Sharon D. et al (2013) A single-molecule long-read survey of the human transcriptome. Nat. Biotechnol., 31, 1009. +. 24108091 10.1038/nbt.2705 PMC4075632

[bty098-B44] Shin S.C. et al (2012) Transcriptomics and comparative analysis of three antarctic notothenioid fishes. Plos One, 7, e43762. 22916302 10.1371/journal.pone.0043762 PMC3420891

[bty098-B45] Sievers F. et al (2011) Fast, scalable generation of high-quality protein multiple sequence alignments using Clustal Omega. Mol. Syst. Biol., 7. 10.1038/msb.2011.75 PMC3261699 21988835

[bty098-B46] Simpson J.T. et al (2009) ABySS: a parallel assembler for short read sequence data. Genome Res., 19, 1117–1123. 19251739 10.1101/gr.089532.108 PMC2694472

[bty098-B47] Su A.I. et al (2002) Large-scale analysis of the human and mouse transcriptomes. Proc. Natl. Acad. Sci. USA, 99, 4465–4470. 11904358 10.1073/pnas.012025199 PMC123671

[bty098-B48] Surget-Groba Y., Montoya-Burgos J.I. (2010) Optimization of de novo transcriptome assembly from next-generation sequencing data. Genome Res., 20, 1432–1440. 20693479 10.1101/gr.103846.109 PMC2945192

[bty098-B49] Tang F.C. et al (2009) mRNA-Seq whole-transcriptome analysis of a single cell. Nat. Methods, 6, 377–U386. 19349980 10.1038/nmeth.1315

[bty098-B50] Tikkanen M.K. et al (2000) Endogenously expressed estrogen receptor and coactivator AIB1 interact in MCF-7 human breast cancer cells. Proc. Natl. Acad. Sci. USA, 97, 12536–12540. 11050174 10.1073/pnas.220427297 PMC18799

[bty098-B51] Tilgner H. et al (2014) Defining a personal, allele-specific, and single-molecule long-read transcriptome. Proc. Natl. Acad. Sci. USA, 111, 9869–9874. 24961374 10.1073/pnas.1400447111 PMC4103364

[bty098-B52] Tilgner H. et al (2015) Comprehensive transcriptome analysis using synthetic long-read sequencing reveals molecular co-association of distant splicing events. Nat. Biotechnol., 33, 736–742. 25985263 10.1038/nbt.3242 PMC4832928

[bty098-B53] Trapnell C. et al (2010) Transcript assembly and quantification by RNA-Seq reveals unannotated transcripts and isoform switching during cell differentiation. Nat. Biotechnol., 28, 511–515. 20436464 10.1038/nbt.1621 PMC3146043

[bty098-B54] Wang Z. et al (2009) RNA-Seq: a revolutionary tool for transcriptomics. Nat. Rev. Genet., 10, 57–63. 19015660 10.1038/nrg2484 PMC2949280

[bty098-B55] Weirather J.L. et al (2015) Characterization of fusion genes and the significantly expressed fusion isoforms in breast cancer by hybrid sequencing. Nucleic Acids Res., 43, e116. 26040699 10.1093/nar/gkv562 PMC4605286

[bty098-B56] Wu T.D., Watanabe C.K. (2005) GMAP: a genomic mapping and alignment program for mRNA and EST sequences. Bioinformatics, 21, 1859–1875. 15728110 10.1093/bioinformatics/bti310

[bty098-B57] Wu Z.G. et al (2016) Insights from the cold transcriptome and metabolome of *Dendrobium officinale*: global reprogramming of metabolic and gene regulation networks during cold acclimation. Front. Plant Sci., 7. 10.3389/fpls.2016.01653 PMC5099257 27877182

[bty098-B58] Xie Y. et al (2014) SOAPdenovo-Trans: de novo transcriptome assembly with short RNA-Seq reads. Bioinformatics, 30, 1660–1666. 24532719 10.1093/bioinformatics/btu077

[bty098-B59] Yan L. et al (2015) The genome of *Dendrobium officinale* illuminates the biology of the important traditional Chinese Orchid Herb. Mol. Plant, 8, 922–934. 25825286 10.1016/j.molp.2014.12.011

[bty098-B60] Zerbino D.R., Birney E. (2008) Velvet: algorithms for de novo short read assembly using de Bruijn graphs. Genome Res., 18, 821–829. 18349386 10.1101/gr.074492.107 PMC2336801

