# Supplementary material for: IDP-denovo: de novo transcriptome assembly and isoform annotation by hybrid sequencing
Source: Bioinformatics. 2018 Feb 23;34(13):2168–76. doi: 10.1093/bioinformatics/bty098 (PMC6022631; doi:10.1093/bioinformatics/bty098)
Supplement: Supplementary Data [file bty098_supplementary_bioinfom_revised_20180226.pdf]

Supplementary Material

Note 1: Algorithm complexity of clustering unaligned LRs

IDP-denovo employs a greedy incremental clustering algorithm for *k*-mer clustering.

First, unaligned long reads (LRs) are processed with homopolymer compression, which has the complexity $O(Nl)$, where N is the number of unaligned LRs and l is the average length of those LRs. Then, LRs are sorted by decreasing lengths with the complexity $O(NlogN)$ using the C++ standard library.

Second, the longest LR is assigned to the first cluster and it is set as the representative LR of this cluster. When considering the next LR, we examine the percentages of shared *k*-mers between this LR and the representative LRs of all existing clusters. Extraction of *k*-mers is with the complexity $O(l)$ for each LR. Bloom filters are used to store and query *k*-mers with the complexity $O(1)$ for either (Melsted and Pritchard, 2011). The worst case for clustering is that each cluster contains only one LR, and thus the shortest LR need to be compared to each representative LR from $N-1$ existing clusters. The time complexity of this step is $O(N^2l)$.

Taken together, the time complexity for clustering unaligned LRs is $O(N^2l)$.

Note 2: Algorithms for pseudo-reference generation

Algorithm 1. Generating_consensus

Input: sequence s, a set of sequences T
Output: consensus sequence p

$V \leftarrow \{s\}$
for each $t \in T$
  if *s and t have* $\geq 30\%$ *identities* **then**
    $V \leftarrow V \cup \{t\}$
  end if
end for
$p \leftarrow$ *consensus generated from members in V via multiple sequence alignment with Clustal Omega*
return p

Algorithm 2. Generating pseudo-reference

Input: a set of sequences L
Output: pseudo-reference m

$N \leftarrow$ *number of input sequences L*
$\{l_1, l_2, \ldots, l_N\} \leftarrow$ *input sequences after sorting by descending order of lengths*
$m \leftarrow l_1$
$i \leftarrow 2$
while $i \leq N$ **do**
  if $i = N$ **then**
    $G \leftarrow \emptyset$
    $G \leftarrow G \cup \{l_i\}$
    $m \leftarrow$ *Generating_consensus*(m, G)
  else
    $G \leftarrow \emptyset$
    $G \leftarrow G \cup \{l_i\}$
    $G \leftarrow G \cup \{l_{i+1}\}$
    $m \leftarrow$ *Generating_consensus*(m, G)
  end if
  $i \leftarrow i + 2$
end while
return m

Figure S1. The algorithms for pseudo-reference generation.

Note 3: Optimization of gap length cutoff for a possible alternative exon usage event

Errors in the assembled transcript sequences can cause small gaps (i.e., indel) in transcript alignment to pseudo-reference sequences. Therefore, we need to find a gap length cutoff to distinguish alternative exon usage events from error-caused gaps.

We investigated the length distribution of alternative exon usage in the Ensembl annotation library (version 79) (Cunningham, et al., 2015). A toy example is shown below (Figure S2). The lengths within gaps between adjacent exons were labeled in black above the gaps. In this example, we can get the lengths of alternative exon usage as 85, 70, 35, 50 and 15 bp. Considering all genes and transcripts in the whole Ensembl annotation library (version 79), we found 95% alternative exon usage were ≥43 bp. Since error rates of LRs and SRs are 10-20% and <1%, respectively, the probability of an error-caused gap ≥43 bp is very low, so we set 43 bp as the gap length cutoff to determine if a gap in alignment indicates an alternative exon usage event.

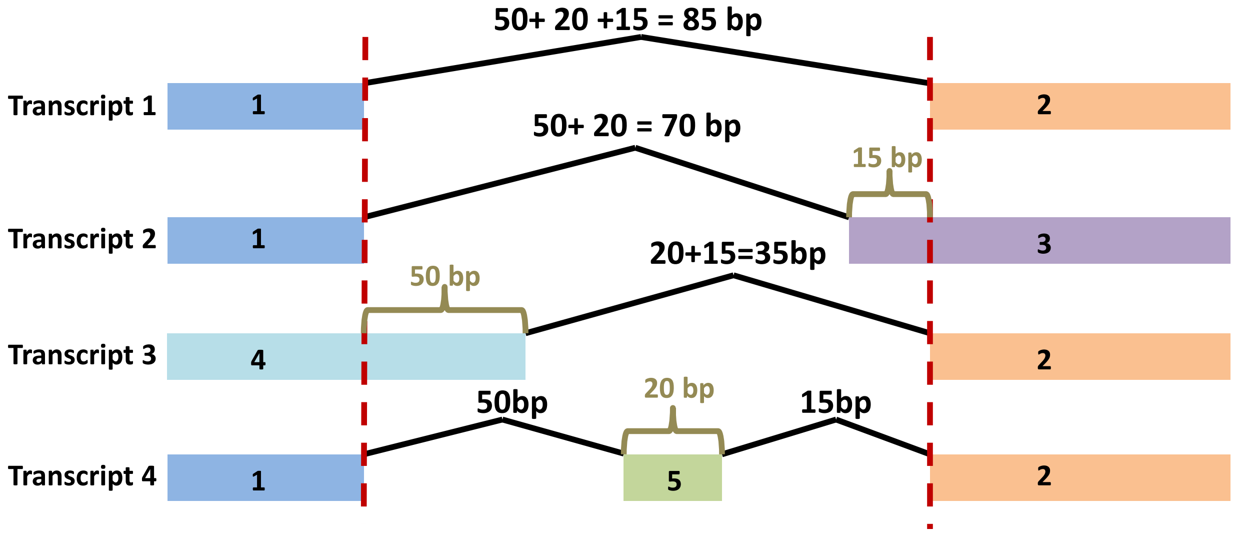

Figure S2. Schematic illustration of computing lengths of alternative exon usage (i.e., genomic regions between adjacent exons that are covered by exons from other transcripts).

Note 4: Dataset for performance evaluation

To assess the performance of IDP-denovo, human RNA-seq data produced on the Illumina and Pacific Biosciences (PacBio) platforms from GM12878 cell line (SRP036136) (Tilgner, et al., 2014) were used. Read quality was checked with FastQC, and 10 bp at the end were trimmed. LRs from the PacBio platform were corrected by short reads (SRs) from the Illumina platform by the error correction tool LSC (Au, et al., 2012).

Note 5: Parameter settings of existing SR-scaffold assembly algorithms and precision-recall statistics

To choose an optimal SR-scaffold assembly algorithm, we applied performance evaluation granularities (Li, et al., 2014) including precision (fraction of matched nucleotides of assembled transcripts), recall (fraction of matched nucleotides of reference transcripts), and F_1 score (harmonic mean of precision and recall) to pick out the SR-alone algorithm with the best performance for SR-scaffold assembly. We tested the existing tools Trinity (version 2.1.1) (Grabherr, et al., 2011), SOAPdenovo-Trans (version 1.03) (Xie, et al., 2014), Bridger (version r2014-12-01) (Chang, et al., 2015), Trans-ABySS (version 1.5.3) (Robertson, et al., 2010), and Oases (version 0.2.9), which is an assembly pipeline with input of preliminary assembly by SRs from Velvet (version 1.2.10) (referred to herein as "Velvet+Oases") (Schulz, et al., 2012; Zerbino and Birney, 2008). Next, we aligned the assembled SR-scaffolds to the reference genome (GRCh38) by GMAP (Wu and Watanabe, 2005), and the precision, recall and F_1 score of each tool were calculated. The length and coverage cutoff of *k*-mer were set as 31 and 10 for SOAPdenovo-Trans, Trans-ABySS, and Velvet+Oases; they were set as defaults for Trinity and Bridger. Velvet+Oases showed the best performance among the five SR-alone methods. Therefore, as the first step of IDP-denovo, *de novo* assembly is applied to SRs by the assembly algorithm Velvet+Oases, with assembly parameters length and coverage cutoff of *k*-mer set as 31 and 10, respectively. PacBio LRs were aligned to the human genome by GMAP, and 697,247 LRs that could be annotated by Ensembl gene annotation (version 79) were used in evaluation. Assembled transcripts that cover all splice sites in annotation are considered as full-length gene isoforms.

Note 6: Comparison of abundances between transcripts covered by SR-scaffolds and missed by SR-scaffolds but covered by LRs

The boxplot below shows differences of their abundances: the transcripts missed by SR-scaffolds but covered by LRs have significantly lower FPKM than those covered by SR-scaffolds (Figure S3, p-value< 2.2e-16). It indicates that LRs can rescue lowly expressed transcripts that are missed by the SR assembly method.

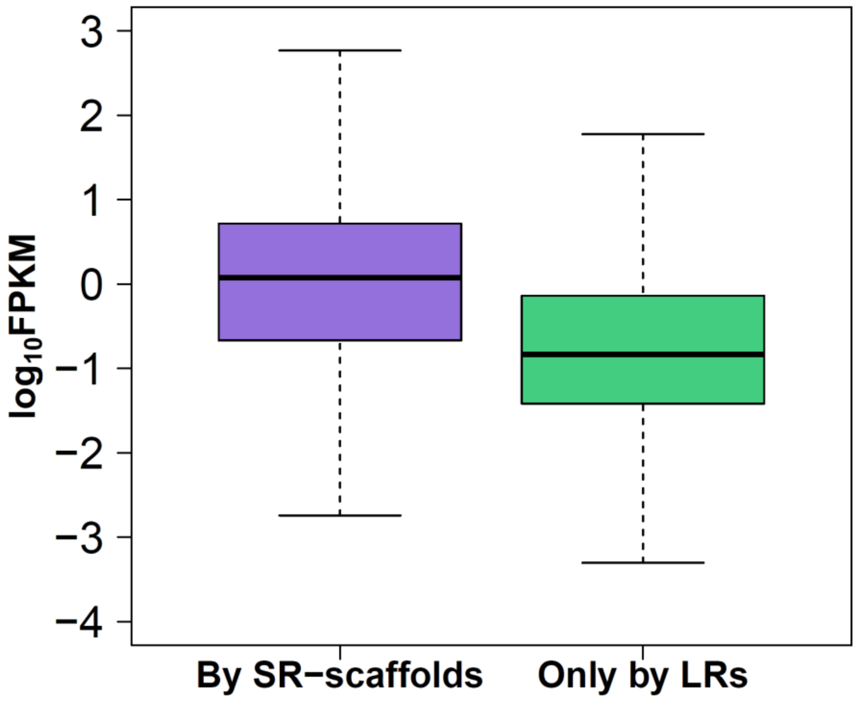

Figure S3. Comparison of abundances between transcripts covered by SR-scaffolds and those missed by SR-scaffolds but covered by LRs. To reduce effects of highly expressed transcripts and to avoid skewed distribution of transcript expression, we log10-tranformed FPKM of transcripts (FPKM ≥ 1e-6) from those covered by SR-scaffolds (80.54%, n= 17,243) and those only by LRs (66.60%, n=13,780), with FPKM computed by StringTie (Pertea, et al., 2015) with SR coverage, then performed t-test between these two groups (Littlejohn, et al., 2014; Xu and Su, 2015; Zwiener, et al., 2014). Outliers are not included. There are some assembled transcripts with unappreciable FPKM: FPKM was estimated by SRs, and thus transcripts only assembled by LRs may have unappreciable FPKM; for very low-expressed SR-assembled transcripts, they may be caused by incorrect SR-assembly or incorrect abundance estimation of FPKM.

Note 7: The influences of SR and LR coverage on assembly accuracy

To investigate the influences of SR and LR coverage, the output transcripts from GM12878 dataset by IDP-denovo were binned according to their SR coverage (estimated by StringTie) and LR coverage (number of aligned LRs) separately, with the roughly equal numbers of transcripts in each bin, and the accuracy metrics at average, including precision, recall and F_1 score, were evaluated (Figure S4).

The transcript accuracy improves with increasing coverage of SRs or LRs.

1) Influence of SR coverage: High SR coverage aids in assembly of accurate SR-scaffolds (step a1 in Figure 1 in main text), while low SR coverage can lead to low-accuracy assembly that further prevents long reads from being aligned correctly to extend SR-scaffolds.
2) Influence of LR coverage: For the regions uncovered by SR-scaffolds, LRs are used for extension to get full-length transcripts (step a3 in Figure 1 in main text). High LR coverage is helpful to generate accurate consensus from error-prone LRs.

Therefore, either high SR or high LR coverage contributes to accurate transcript assembly by IDP-denovo.

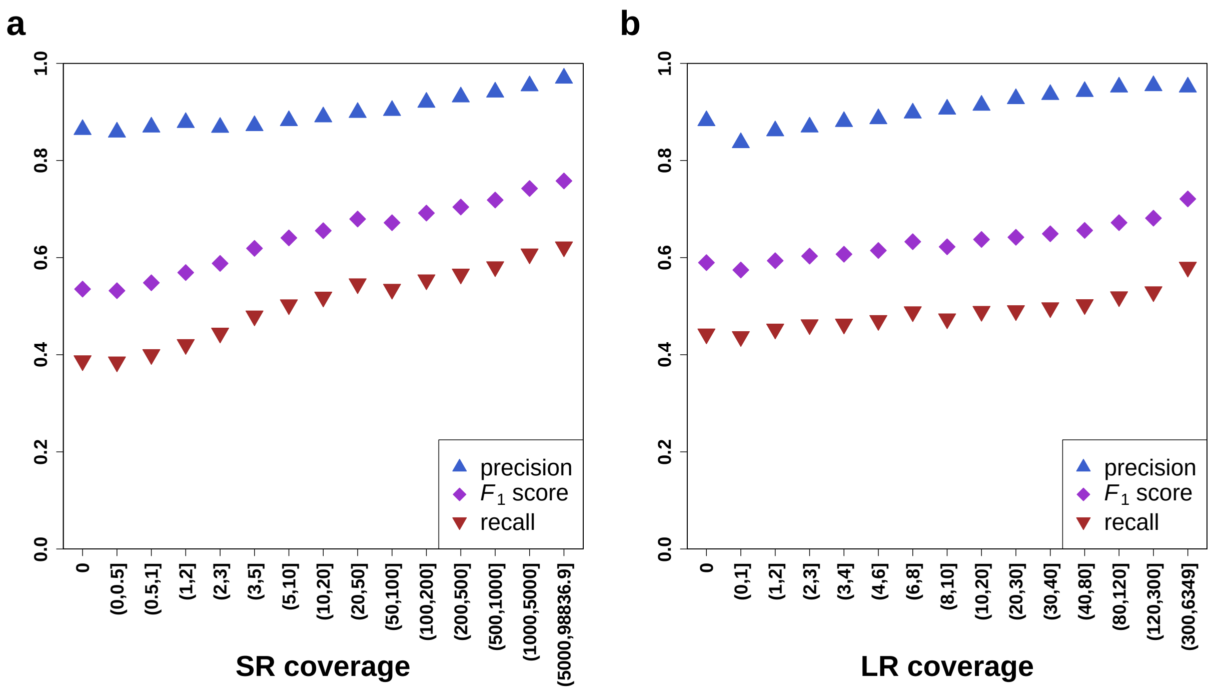

Figure S4. The influences of SR and LR coverage on assembly accuracy, with metrics of precision, recall and F_1 score.

Note 8: Parameter settings of existing assembly methods with Hybrid-Seq data and evaluation granularities

Two existing assembly methods with Hybrid-Seq data were tested: 1) Trinity (version 2.1.1), which can integrate LRs into *de novo* assembly on SRs to improve assembly of isoforms with complex structures, and 2) a hybrid *de novo* transcriptome assembly pipeline proposed by the Roulin group (referred to herein as “Roulin’s pipeline”) (Roulin, et al., 2014), which assembles Roche 454 LRs and Illumina SRs separately, followed by clustering and removal of redundant contigs with usearch (Edgar, 2010) and CAP3 (Huang and Madan, 1999). Parameters were set as default for these two methods. The performance of IDP-denovo was compared to these two existing assembly methods by

GM12878 data with precision-recall statistics (Li, et al., 2014) mentioned above and sensitivity (the number of reconstructed full-length transcripts) that were described in the previous study (Chang, et al., 2015).

Note 9: Evaluation strategies for *k*-mer clustering

To optimize the performance of the *k*-mer-based clustering method, 94,506 LRs from the GM12878 (Tilgner, et al., 2014) dataset that were annotated with genes in Chr19 (chromosome 19) in Ensembl database by alignment with GMAP, were used as training data. Four typical measures of clustering performance, including the Jaccard Index, precision, recall, and F-measure, were applied (Bao, et al., 2011; Chen, et al., 2006). Let *a* be the number of pairs that are from the same class and grouped into the same cluster. Let *b* be the number of pairs that are from the same class but grouped into different clusters. Let *c* be the number of pairs that are from different classes but grouped into the same cluster. The Jaccard Index is computed as $a/(a+b+c)$. Precision is computed as $a/(a+c)$ and recall as $a/(a+b)$. F-measure is computed as 2x precision/(precision+recall).

The optimal values of these measures were obtained when $k = 15$ and $C_{threshold} = 0.05$ for all LRs from chr19 as well as unaligned LRs from chr19 (Table S1). Therefore, these parameter settings were used to cluster unaligned LRs.

Table S1. Performance of *k*-mer clustering with different combinations of lengths of *k*-mer and percentage cutoff $C_{threshold}$ with unaligned LRs from chr19.

	**Percentage cutoff**	**Length of *k*-mer**		
	$C_{threshold}$	**13**	**15**	**17**
Jaccard index	0.04	0.991	***<u>0.992</u>***	***<u>0.992</u>***
	0.05	***<u>0.992</u>***	***<u>0.992</u>***	0.987
	0.06	***<u>0.992</u>***	0.987	0.987
Precision	0.04	0.998	0.999	0.999
	0.05	0.999	0.999	0.999
	0.06	0.999	0.999	***<u>1.000</u>***
Recall	0.04	0.993	0.993	***<u>0.994</u>***
	0.05	0.993	***<u>0.994</u>***	0.988
	0.06	***<u>0.994</u>***	0.988	0.987
F-measure	0.04	***<u>0.996</u>***	***<u>0.996</u>***	***<u>0.996</u>***
	0.05	***<u>0.996</u>***	***<u>0.996</u>***	0.994
	0.06	***<u>0.996</u>***	0.994	0.993

[a] Results with the best performance for each performance measure are bold, underlined and italic.

Note 10: Evaluation strategy for annotation of gene isoform structures

The annotation analysis was applied to clusters with at most 30 sequences, which comprised of 89.67% of all clusters. To examine the accuracy of isoform structure annotation, transcript sequences from a cluster were aligned to the reference genome by GMAP. A gap in alignment is supposed to be an alternative exon usage event. The 5' end splice site from the reported skipped exon corresponds to the nearest annotated 5' end splice site in alignment. Identification error is defined as the difference between the positions of the predicted 3' end splice site by IDP-denovo and the 3' end splice site reported by reference-alignment.

Note 11: Comparison to abundance estimated by StringTie

SR and LR abundance indices reported by IDP-denovo were compared to FPKM reported by StringTie (Pertea, et al., 2015) for each annotated isoform on a natural-logarithmic scale, if all these values were positive. 5,967 isoforms were included. We calculated Spearman and Pearson correlation coefficients between the SR abundance index and the FPKM estimated by StringTie, as well as those between LR abundance index and FPKM estimated by StringTie.

Note 12: Application of IDP-denovo to *Dendrobium officinale*

To demonstrate application of IDP-denovo to non-model organisms, Illumina and PacBio data of *D. officinale* were used (accession number SRP094520). Read quality was checked with FastQC, and 13 bp at the end were trimmed. LRs from the PacBio platform were corrected by SRs from the Illumina platform by LSC. The two SR-scaffold assembly parameters of Velvet+Oases, length and coverage cutoff of *k*-mer, were set as 31 and 10, respectively. A previously published annotation library and a draft assembly of *D. officinale* genome (Yan, et al., 2015), polished by an assembled transcriptome (Wu, et al., 2016), were used to evaluate the IDP-denovo output. Annotation was performed by alignment of assembled sequences to the draft assembly by GMAP with aligned sequences with minimal alignment length of 30 nts were annotated, while the best alignment reported no overlap to annotated loci, the second best alignment was considered if the alignment length was at least 70% of the best

alignment. Transcripts unaligned to annotated loci in the draft genome were considered as novel transcripts from novel genes.

Supplementary references

FastQC: A quality control tool for high throughput sequence data. [http://www.bioinformatics.babraham.ac.uk/projects/fastqc/].

Au, K.F., *et al.* Improving PacBio long read accuracy by short read alignment. *PLoS One* 2012;7(10):e46679.

Bao, E., *et al.* SEED: efficient clustering of next-generation sequences. *Bioinformatics* 2011;27(18):2502-2509.

Chang, Z., *et al.* Bridger: a new framework for de novo transcriptome assembly using RNA-seq data. *Genome Biology* 2015;16.

Chen, Y.H., *et al.* SEQOPTICS: a protein sequence clustering system. *Bmc Bioinformatics* 2006;7.

Cunningham, F., *et al.* Ensembl 2015. *Nucleic Acids Res* 2015;43(D1):D662-D669.

Edgar, R.C. Search and clustering orders of magnitude faster than BLAST. *Bioinformatics* 2010;26(19):2460-2461.

Grabherr, M.G., *et al.* Full-length transcriptome assembly from RNA-Seq data without a reference genome. *Nat Biotechnol* 2011;29(7):644-U130.

Huang, X.Q. and Madan, A. CAP3: A DNA sequence assembly program. *Genome Res* 1999;9(9):868-877.

Li, B., *et al.* Evaluation of de novo transcriptome assemblies from RNA-Seq data. *Genome Biology* 2014;15(12).

Littlejohn, M.D., *et al.* Expression Variants of the Lipogenic AGPAT6 Gene Affect Diverse Milk Composition Phenotypes in Bos taurus. *Plos One* 2014;9(1).

Melsted, P. and Pritchard, J.K. Efficient counting of k-mers in DNA sequences using a bloom filter. *Bmc Bioinformatics* 2011;12.

Pertea, M., *et al.* StringTie enables improved reconstruction of a transcriptome from RNA-seq reads. *Nat Biotechnol* 2015;33(3):290-+.

Robertson, G., *et al.* De novo assembly and analysis of RNA-seq data. *Nature Methods* 2010;7(11):909-U962.

Roulin, A.C., *et al.* De Novo Transcriptome Hybrid Assembly and Validation in the European Earwig (Dermaptera, Forficula auricularia). *Plos One* 2014;9(4).

Schulz, M.H., *et al.* Oases: robust de novo RNA-seq assembly across the dynamic range of expression levels. *Bioinformatics* 2012;28(8):1086-1092.

Tilgner, H., *et al.* Defining a personal, allele-specific, and single-molecule long-read transcriptome. *P Natl Acad Sci USA* 2014;111(27):9869-9874.

Wu, T.D. and Watanabe, C.K. GMAP: a genomic mapping and alignment program for mRNA and EST sequences. *Bioinformatics* 2005;21(9):1859-1875.

Wu, Z.G., *et al.* Insights from the Cold Transcriptome and Metabolome of Dendrobium officinale: Global Reprogramming of Metabolic and Gene Regulation Networks during Cold Acclimation. *Front Plant Sci* 2016;7.

Xie, Y., *et al.* SOAPdenovo-Trans: de novo transcriptome assembly with short RNA-Seq reads. *Bioinformatics* 2014;30(12):1660-1666.

Xu, C. and Su, Z.C. Identification of cell types from single-cell transcriptomes using a novel clustering method. *Bioinformatics* 2015;31(12):1974-1980.
Yan, L.*, et al.* The Genome of Dendrobium officinale Illuminates the Biology of the Important Traditional Chinese Orchid Herb. *Mol Plant* 2015;8(6):922-934.
Zerbino, D.R. and Birney, E. Velvet: Algorithms for de novo short read assembly using de Bruijn graphs. *Genome Res* 2008;18(5):821-829.
Zwiener, I., Frisch, B. and Binder, H. Transforming RNA-Seq Data to Improve the Performance of Prognostic Gene Signatures. *Plos One* 2014;9(1).
